# Systemic lupus erythematosus and granulomatous lymphadenopathy

**DOI:** 10.1186/1471-2431-13-179

**Published:** 2013-11-05

**Authors:** Devendra Shrestha, Ajaya Kumar Dhakal, Shiva Raj KC, Arati Shakya, Subhash Chandra Shah, Henish Shakya

**Affiliations:** 1 Department of Pediatrics, KIST Medical College Teaching Hospital, Imadol, Lalitpur, Nepal; 2 Department of Pathology, KIST Medical College Teaching Hospital, Imadol, Lalitpur, Nepal

**Keywords:** Granulomatous, Lymphadenopathy, SLE

## Abstract

**Background:**

Systemic lupus erythematosus (SLE) is known to present with a wide variety of clinical manifestations. Lymphadenopathy is frequently observed in children with SLE and may occasionally be the presenting feature. SLE presenting with granulomatous changes in lymph node biopsy is rare. These features may also cause diagnostic confusion with other causes of granulomatous lymphadenopathy.

**Case presentation:**

We report 12 year-old female who presented with generalized lymphadenopathy associated with intermittent fever as well as weight loss for three years. She also had developed anasarca two years prior to presentation. On presentation, she had growth failure and delayed puberty. Lymph node biopsy revealed granulomatous features. She developed a malar rash, arthritis and positive ANA antibodies over the course of next two months and showed WHO class II lupus nephritis on renal biopsy, which confirmed the final diagnosis of SLE. She was started on oral prednisolone and hydroxychloroquine with which her clinical condition improved, and she is currently much better under regular follow up.

**Conclusion:**

Generalized lymphadenopathy may be the presenting feature of SLE and it may preceed the other symptoms of SLE by many years as illustrated by this patient. Granulomatous changes may rarely be seen in lupus lymphadenitis. Although uncommon, in children who present with generalized lymphadenopathy along with prolonged fever and constitutional symptoms, non-infectious causes like SLE should also be considered as a diagnostic possibility.

## Background

Generalized lymphadenopathy along with fever is commonly encountered in pediatric clinical practice. Infections, malignancy and connective tissue diseases are diverse groups of illnesses causing generalized lymphadenopathy with fever. The majority of these are infectious in origin and may be self limiting [1]. Although not included in the American College of Rheumatology (ACR) diagnostic criteria for systemic lupus erythematosus (SLE), generalized lymphadenopathy is frequently observed in children with SLE and may be the presenting feature in the absence of other clinical manifestations. This may pose a diagnostic dilemma, and therefore a lymph node biopsy is warranted in this subset of patients.

The exact etiology of SLE is still unclear, although multifactorial interaction with genetic and environmental factors has been implicated. It is characterized by the formation of autoantibodies to various components of the cell nucleus leading to inflammation, vasculitis and immune complex deposition. Immune complex deposition along with complement activation has been postulated for various manifestations of SLE including lupus nephritis, which is also demonstrated by frequent association of hypocomplementemia and signs of vasculitis at the sites of active SLE [2].

Few early reports have described non-caseating epithelioid cell granulomas in necropsy specimens of serous membranes, lung, lymph node, and spleen [3,4] as well as pleural biopsy specimen of a patient with SLE [5]. Granuloma formation in SLE is rare and the pathogenesis is unclear. Here in, we report an adolescent south Asian female presenting with generalized lymphadenopathy with granulomatous features with a final diagnosis of SLE.

## Case presentation

A 12 year-old girl presented to KIST Medical College Teaching Hospital in 2011 with complaints of painless lymph node swelling in bilateral neck and axillae for three years, along with a history of weight loss, generalized weakness, and fever. However, there was no history of joint pain, skin rash, edema, hematuria, or bone pain at the presentation. There was no contact history of tuberculosis, and there was no history of similar illness or of autoimmune diseases in the family.

In 2008, she was evaluated at another hospital for lymphadenopathy which showed reactive changes in fine needle aspiration cytology (FNAC) and no further treatment was instituted. She had developed generalized swelling of her body in 2009 for which she was evaluated at a different institute and improved after taking oral medications for one month. However, detailed medical records were not available.

On examination, she was febrile, BP 100/60 mm Hg and was pale. There were multiple enlarged lymph nodes in both cervical, axillary and inguinal regions which were soft, non tender and discrete with the largest measuring 5 cm × 5 cm in diameter. She had hepatosplenomegaly, but there was no edema, skin rash, or bone tenderness. Her BMI was 14.03 (below 3^rd^ percentile) and she was in prepubertal SMR stage.

**Figure 1:**
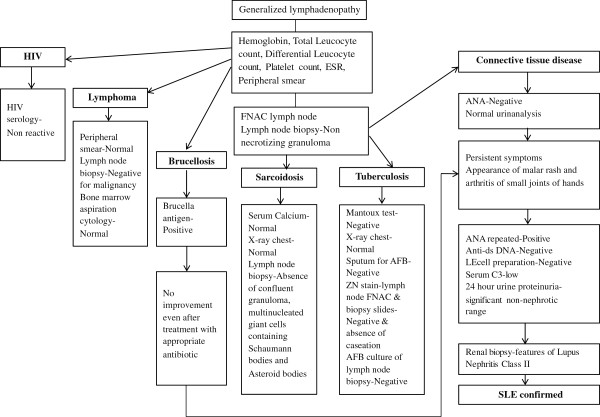
Flow diagram of diagnostic work up.

She was evaluated keeping diagnostic possibilities of tuberculosis, HIV, connective tissue disease, lymphoma, and sarcoidosis as shown in Figure 1. Her investigations revealed as follow (Table 1).

**Table 1:** Investigations

	**2011/11/26**	**2012/01/31**
Hemoglobin	9.2 gm/dl	10.5 gm/dl
TLC	6300/mm^3^	6800/mm^3^
DLC	N70 L28 E2	N 81 L19
Platelet	478000/mm^3^	359000/mm^3^
ESR	61 mm at 1 hour	56 mm at 1 hour
Peripheral smear	RBC predominantly normocytic normochromic, no abnormal cells	
Urinanalysis	Albumin trace, no RBC	Albumin trace, no RBC
Mantoux test	No induration after 72 hours	
CRP	Positive	
Rheumatoid factor	Negative	
HIV serology	Non reactive	
ANA	Negative	Positive
Anti-dsDNA antibody	Negative	23.4 U/L (Negative <30.0 U/L)
Anti U1SNRNP antibody		Negative
LE cell preparation		Negative
Serum C3		87 mg/dl (84–168 mg/dl)
24 hour urinary protein		35.8 mg/m^2^/hour
VDRL		Non reactive
Brucella Antigen	Positive	
Serum Calcium	9.2 mg/dl	

Abdominal ultrasonography showed multiple mesenteric lymph nodes, the largest measuring 17 mm × 10 mm in diameter with no ascites. Her chest x-ray and bone marrow aspiration cytology were normal.

**Figure 2:**
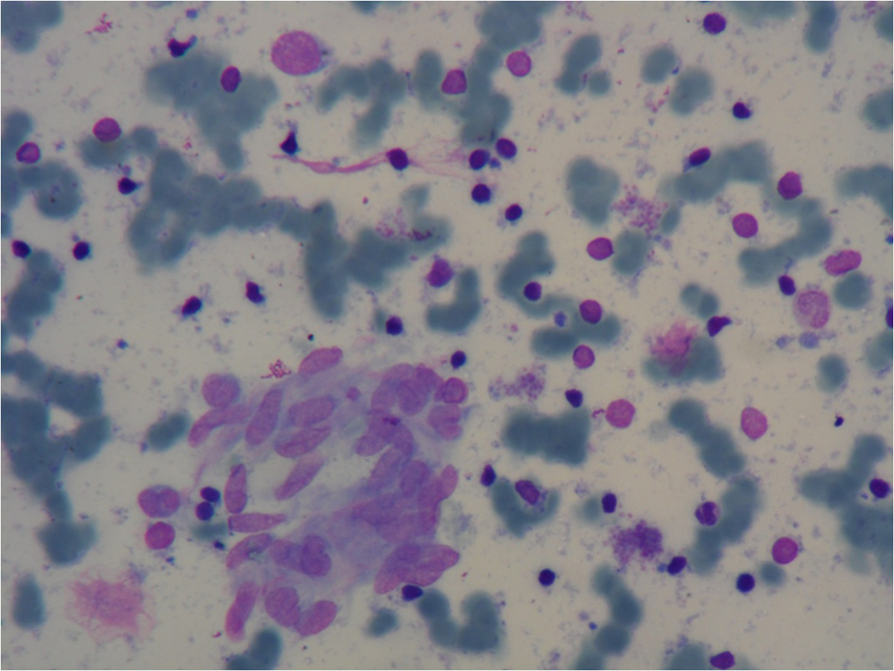
**Photomicrograph of FNAC of a cervical lymph node.** FNAC smear showing clusters of epithelioid cells in a lymphoid background (Wright stain, X100).

**Figure 3:**
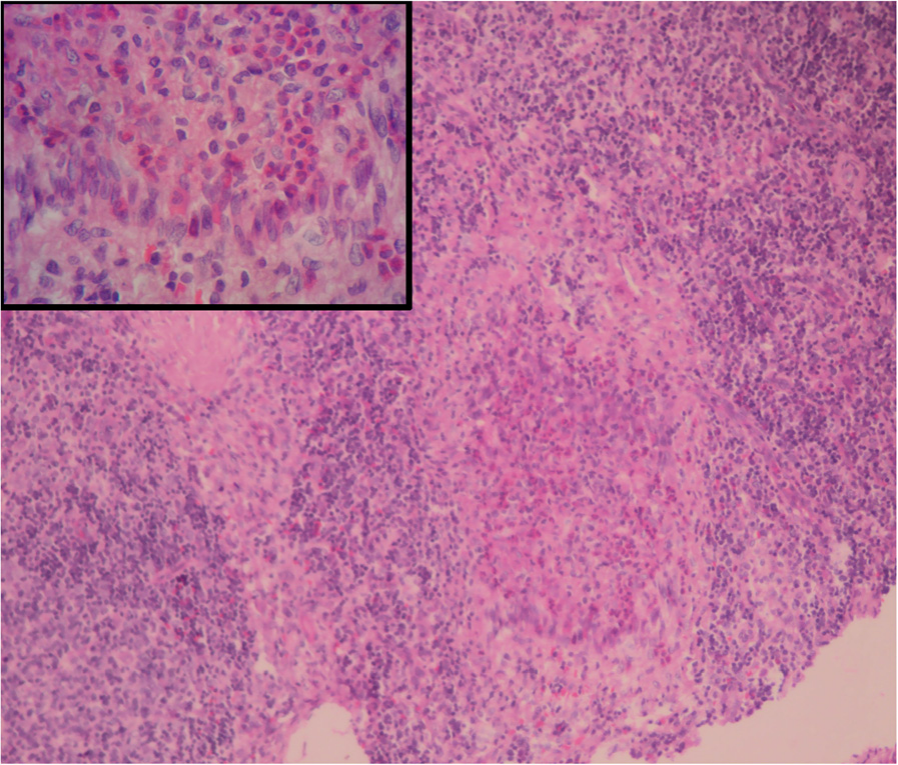
**Photomicrograph of a cervical lymph node biopsy.** Lymph node biopsy showing epithelioid granuloma admixed with eosinophils, lymphocytes and plasma cells (H&E stain, X40). Inset showing epithelioid granuloma in higher magnification (H&E stain, X400).

FNAC of cervical lymph node was suggestive of granulomatous lymphadenopathy (Figure 2). Excisional biopsy of cervical lymph node on H & E staining showed intact capsule and lymph nodal architecture was partially effaced by epithelioid granuloma admixed with several eosinophils, lymphocytes and plasma cells. Other areas showed lymphoid follicles with prominent germinal center. The paracortical area showed an exaggeration of high endothelial venules (Figure 3). Ziehl Neelsen staining as well as Acid Fast Bacilli (AFB) culture of the lymph nodes was negative. Immunohistochemistry study of lymph node was not available.

**Figure 4:**
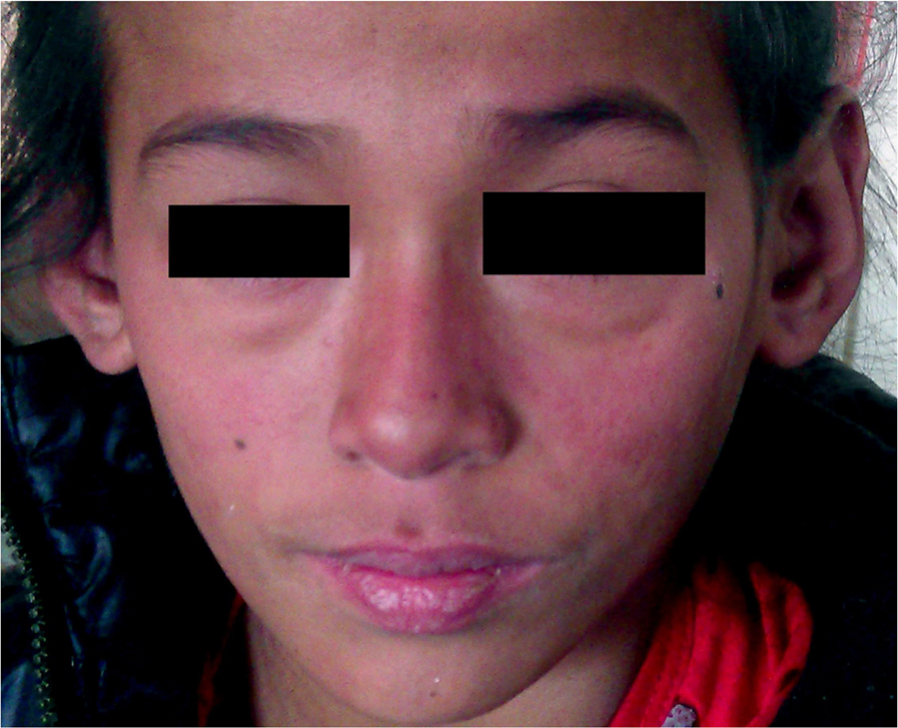
Photograph showing erythematous malar rash sparing nasolabial fold.

She was suspected to have brucellosis on the basis of serology and was treated accordingly. However, she continued to have fever and persistent lymphadenopathy. She was admitted in the pediatric ward several times over a two months period. During the last hospital stay, she developed a malar rash extending to nasal bridge sparing nasolabial fold along with arthritis of small joints of both hands (Figure 4).

On renal biopsy, there was normal cellularity, absence of sclerosis, crescents and blood vessel thickening in light microscopy. IgA and IgM autofluorescence in tubular casts, C3 autofluorescence in blood vessels were seen in immunofluorescence microscopy which suggested the diagnosis of lupus nephritis (pure mesangial alterations), WHO class II. Anti-Sm antibody level was not available.

Thus the final diagnosis of SLE was made and treatment was started with oral prednisolone and hydroxychloroquine. Malar rash disappeared a few days after initiation of steroid treatment. On subsequent follow up after two weeks, lymph nodes size drastically reduced, hepatosplenomegaly size was decreased, and constitutional symptoms had also improved. She was kept under close follow up on maintenance prednisolone. However, on subsequent follow up, she had persistent non-nephrotic range proteinuria (17.5 mg/m^2^/hour), low C3 level (42.3 mg/dl) and high ESR without flaring up of other symptoms. After ruling out noncompliance to steroid therapy, possibility of progression of lupus nephritis or incorrect initial staging was suspected. Hence, repeat renal biopsy was considered in March 2013 and histopathology showed features of glomerulopathy exhibiting segmental mild increase in mesangial cellularity and matrix. Direct immunofluorescence studies revealed mesangial granular staining with dominance of IgA, and similar less intense staining for other immunoglobulins and light chain components. Staining for C3 and C1q was weak in glomerular mesangial areas. This was consistent with mesangial lupus nephritis, Class II (ISN/RPS). She is presently on maintenance prednisolone and on regular follow up.

## Conclusions

SLE is a chronic autoimmune disorder which is characterized by inflammation of blood vessels and connective tissue involving multiple systems and presence of circulating autoantibodies especially ANA and Anti-dsDNA antibodies. SLE is relatively uncommon in children and estimated incidence ranges from 10 to 20 per 100 000 children depending on the ethnic population [6].

Children and adolescents represent 15-20% of all patients with SLE [7]. Females are affected three to five times more often than males [8]. SLE is well known to present with a wide variety of clinical manifestations. Children with SLE may present with constitutional symptoms (fatigue, myalgia, weakness, weight loss), skin rashes, especially malar rash or arthralgia/arthritis that may precede the detection of organ-specific lesion [8].

Prevalence of lymphadenopathy has been reported in 23-34% of patients with SLE [9] with a female preponderance [10]. Despite being frequent manifestation, lymphadenopathy is not included in the ACR criteria for the diagnosis of SLE. Lymphadenopathy has even been reported as the first clinical manifestation of SLE in children [11-14]. Lymphadenopathy is very common in children due to variety of etiologies and hence may pose a delay in the diagnosis of SLE.

Lymph nodes in SLE are usually soft, non tender, mobile, generalized and of varying size [15,16]. We also observed similar findings in our patient. On histopathology, coagulative necrosis with hematoxylin bodies is specific to lupus lymphadenitis but is rarely observed [10]. More commonly lymph node biopsies in SLE show reactive follicular hyperplasia which is considered a nonspecific feature. Atypical lymphoproliferation found in the lymph node biopsies of SLE were classified as reactive follicular hyperplasia with giant follicles, aspects similar to Castelman’s disease, atypical paracortical hyperplasia with lymphoid follicles, and atypical immunoblastic and lymphoplasmacytic proliferation [10]. A classification system with three patterns of histopathological features of lupus lymphadenitis had been proposed, however they did not appear to be highly specific for SLE [17]. The occurrence of variety of histopathological features poses diagnostic difficulty and should be differentiated from lymphoproliferative disorders. This patient had undergone bone marrow examination which was normal.

The histopathological feature of lymph node in our patient was epithelioid granuloma admixed with several eosinophils, lymphocytes and plasma cells along with other areas showing features of reactive lymphadenitis. Earlier reports described non-caseating epithelioid cell granulomas consisting of central zone of epithelioid cells surrounded by dense infiltrate of histiocytes without giant cells in necropsy specimens of patients with SLE [3,4]. Similarly several granulomas consisting of large epithelioid cells and histiocytes with granular and fibrinoid appearance in the center without caseation were observed in a pleural biopsy of a patient with SLE [5]. The presence of granuloma in lymph node biopsy and FNAC is suggestive of tuberculosis which is highly prevalent in our geographical region. However there was no caseous necrosis or Langhan type of multinucleated giant cells that are usually seen in tubercular lymph node histopathology. Furthermore, Ziehl Neelsen stain for AFB and AFB cultures were negative in this patient. Hence tuberculosis was considered unlikely and sequential evolution of features confirmed SLE in this patient. However, there are cases where anti-tubercular treatment has been initiated in patient with lupus lymphadenopathy when there was a sequential absence of symptoms [14]. Lupus nephritis is considered a risk factor for tuberculosis and some patients develop disseminated tuberculosis even before use of corticosteroids [18]. This patient did not receive anti-tubercular treatment and did not develop flare up of symptoms in spite of corticosteroid treatment. Therefore, the possibility of tuberculosis was considered highly unlikely in this patient.

With the presence of a non-caseating granuloma, the other possible diagnosis was sarcoidosis. However, serum calcium level was normal and confluent granulomas, multinucleated giant cells containing Schaumann bodies, and Asteroid bodies were not seen in this patient [19]. Skin biopsy was not considered as malar rash in this patient was very typical of SLE. In addition, renal biopsy also confirmed lupus nephritis.

Pathogenesis of granuloma formation in SLE is still unclear. It was regarded as a response to tissue injury and considered as a manifestation of allergic tissue reaction [3]. Although SLE is considered type III hypersensitivity reaction which has been extensively studied in patients with lupus nephritis, type IV mediated mechanism also may play a role in autoimmune nephritis [20]. Animal studies revealed presence of high levels of expression of IFN-γ and IL-12, as well as exacerbation of lupus disease after administration of IFN-γ to NZB/W F1 mice [21-23].

Defective clearance of apoptotic bodies by the complement system along with a reduced number of macrophages and capacity for phagocytosis lead to a high level of apoptotic bodies in patients with SLE [24-26]. Hence, persistence of these apoptotic bodies may stimulate granuloma formation.

Formation of granuloma occurs in response to a persistent stimulus and has also been linked with TNF and induction of host matrix metalloproteinase (MMPs) production in tuberculosis [27]. Animal studies showed proliferative glomerulonephritis was associated with infiltrating kidney macrophages, renal expression of IFN-inducible genes and MMPs which were mediated IFN [28]. Granuloma formation in SLE may be due to persistence of apoptotic bodies and/or various cytokines and MMPs. It may also support the role of type IV mediated injury in the pathogenesis of SLE. However, detailed studies are needed.

The association of lymphadenopathy with severe disease activity, more constitutional symptoms as well as hepatomegaly and splenomegaly has been correlated in patients with SLE [9], which was also noted in this patient.

All the symptoms of SLE required to fulfill ACR criteria may not appear at the same time, and it took almost three years from initial presentation of generalized lymphadenopathy to reach the final diagnosis and institute definite treatment for SLE in this patient. Hence, strong clinical suspicion and close follow up is essential for early diagnosis and treatment as well as to improve outcomes in children with SLE. In conclusion, generalized lymphadenopathy may be the presenting feature of SLE and it may preceed other symptoms of SLE by many years as illustrated by this patient. Reactive follicular hyperplasia is commonly observed on histopathology of lymph nodes in SLE, and on rare occasions, there may be presence of granulomas in lymph node biopsies. Pathogenesis of this granuloma formation is still unclear and may possibly support the role of type IV reaction playing an additional role in the pathogenesis of SLE. Therefore, in children who present with generalized lymphadenopathy along with prolonged fever and constitutional symptoms, though uncommon, non-infectious causes like SLE should also be considered as a diagnostic possibility.

### Consent

Written informed consent was obtained from the parent of the patient for publication of this Case Report and any accompanying images. A copy of the written consent is available for review by the Editor-in-Chief of this journal.

## Abbreviations

ACR: American College of Rheumatology; AFB: Acid fast bacilli; ANA: Anti nuclear antibody; BMI: Body mass index; FNAC: Fine needle aspiration cytology; IFN-γ: Interferon gamma; IL-12: Interleukin 12; ISN/RPS: International Society of Nephrology/Renal Pathology Society; MMPs: Matrix metalloproteinases; SLE: Systemic lupus erythematosus; SMR: Sexual maturity rate; WHO: World Health Organization.

## Competing interest

The authors declare that they have no competing interests. The authors received no financial support for the research and/or authorship of this article.

## Authors’ contributions

DS conceived of presenting the report, participated in design and coordination, searched literature and wrote initial draft of the manuscript. AKD helped in literature search and drafting of the manuscript. SRKC helped in reviewing histopathology slides and drafting the manuscript. AS helped in drafting the manuscript and literature search. SCS was involved in draft of manuscript. HS helped to draft the manuscript. DS, AKD, AS, SCS, and HS were involved in the treatment of the patient. All the authors read and approved the final manuscript.

## Pre-publication history

The pre-publication history for this paper can be accessed here:

http://www.biomedcentral.com/1471-2431/13/179/prepub
